# A Reassessment of the SIDS Back to Sleep Campaign

**DOI:** 10.1100/tsw.2005.71

**Published:** 2005-07-21

**Authors:** Ralph Pelligra, Glen Doman, Gerry Leisman

**Affiliations:** ^1^ Ames Research Center, National Aeronautics and Space Administration (NASA), Moffett Field, CA 94035, USA; ^2^ The Institutes for the Achievement of Human Potential, Wyndmoor, PA 19038, USA; ^3^ Carrick Institute for Clinical Ergonomics, Rehabilitation and Applied Neuroscience, School of Engineering Technologies, State University of New York, College at Farmingdale, Lupton Hall, 2350 Broadhollow Road, Farmingdale, NY 11735, USA

**Keywords:** SIDS, public health, medical research, epidemiology, public health, child health, infancy, United States

## Abstract

The Back to Sleep Campaign was initiated in 1994 to implement the American Academy of Pediatrics' (AAP) recommendation that infants be placed in the nonprone sleeping position to reduce the risk of the Sudden Infant Death Syndrome (SIDS). This paper offers a challenge to the Back to Sleep Campaign (BTSC) from two perspectives: (1) the questionable validity of SIDS mortality and risk statistics, and (2) the BTSC as human experimentation rather than as confirmed preventive therapy.

The principal argument that initiated the BTSC and that continues to justify its existence is the observed parallel declines in the number of infants placed in the prone sleeping position and the number of reported SIDS deaths. We are compelled to challenge both the implied causal relationship between these observations and the SIDS mortality statistics themselves.

## INTRODUCTION

Medical advice that stems from epidemiological research can have a profound effect on public health because of the many individuals affected. This applies to false as well as correct advice. It is for this reason that we should attempt continually to identify the benefits or the harm of preventive measures and to question the premises on which they are based. Few would argue with this. In practice, however, there is often resistance to change and a reluctance to believe data that contradict previously held beliefs[1].

The "Back to Sleep Campaign" (BTSC) was initiated in 1994 to implement the American Academy of Pediatrics' (AAP) recommendation that infants be placed in the nonprone sleeping position to reduce the risk of the Sudden Infant Death Syndrome(SIDS)[2]. This paper offers a challenge to the BTSC from two perspectives: (1) the questionable validity of SIDS mortality and risk statistics, and (2) the BTSC as human experimentation rather than as confirmed preventive therapy.

## The Questionable Validity of SIDS Mortality and Risk Statistics

The principal argument that initiated the BTSC and that continues to justify its existence is the observed parallel declines in the number of infants placed in the prone sleeping position and the number of reported SIDS deaths[3,4]. We are compelled to challenge both the implied causal relationship between these observations and the SIDS mortality statistics themselves.

SIDS mortality data derive exclusively from epidemiological studies and both the AAP recommendation and the BTSC were launched primarily on the basis of such reports from various countries outside the U.S. However, while it is axiomatic that the validity of any epidemiological study is critically dependent on diagnostic accuracy, there exists today no global consensus on a definition of SIDS. For example, Sawaguchi et al.[5] acknowledge that “a SIDS diagnosis is not uniform throughout Japan and such a diagnosis is not made based on any internationally recognized definition.” Official views concerning a SIDS diagnosis in Japan differ among pediatricians, legal scholars of forensic medicine, and pathologists. In the same reference, they note that in Scotland in the 1980s, many cases of sudden unexpected infant death (SUD) were reported as “SIDS”, whereas in the 1990s there was a trend reversal and the majority of similar cases were classified as “unknown”.

In 1999, 5 years after the start of the BTSC, Cote et al.[6], in a study of 623 cases of SUD in infancy, found that the percentage of non-SIDS diagnoses was much higher for autopsies performed in a center with expertise in pediatric pathology than in a general hospital or medico-legal institute. Non-SIDS diagnoses were also much higher at age ranges atypical (12–18 months) as compared with typical (1–6 months) for SIDS.

It is reasonable, in light of the above, to question the validity of SIDS prevalence rates that emerge from diverse clinics in diverse countries with varying degrees of expertise in pediatric pathology and with inconsistent diagnostic criteria. One might ask to what extent can the decreasing SIDS rate of the past decade be attributable to increased awareness and improved methodologies and procedures for distinguishing SIDS and non-SIDS deaths and changing diagnostic criteria[7,8]? To what extent does the propensity to assign a diagnosis of SIDS to infants who die during the known vulnerable period between 1 and 6 months, or who are found in the prone position, weaken epidemiological analysis?

Paris et al.[9] attempted to address the concern that decrease in SIDS may be a reflection of changes in diagnostic criteria. They reported that alternative diagnostic criteria did not increase over the period studied (1985–1995, excluding 1991). However, they reviewed only deaths attributed to aspiration, suffocation, or positional asphyxia and make no mention of the number of diagnoses, if any, that were documented by death scene investigations. Changes in alternative diagnoses such as infection, cardiovascular abnormalities, or metabolic disease, which may not show serious symptoms prior to death[6], were not considered. In support of the notion that alternative diagnoses may have increased is the observation that while the reported cases of SIDS decreased from 1999–2001, the overall postneonatal mortality remained stable[10].

There is another important variable that must be taken into account when attempting to establish the validity of SIDS mortality statistics. In 1991, an expert panel convened by the U.S. National Institute of Child Health and Human Development (NICHD)[11] expanded the criteria for establishing a SIDS diagnosis to include a thorough coroner’s investigation of the death scene and a review of the infant’s clinical history in addition to the performance of a complete autopsy. This was a critical change, because cases of suffocation due to unsafe sleeping environments or to infanticide could not be distinguished from SIDS by autopsy alone. Prior to the NICHD recommendation in 1991, the annual rate of SIDS in the U.S. was stable at 1.3–1.4 per 1,000 live births.

Fig. 1 shows that the number of reported SIDS deaths was already in decline prior to the issuance, in June 1992, of the AAP recommendation to place infants in the supine sleeping position. By the time the BTSC was begun in 1994, the prevalence rate that year had already dropped to 1.03 SIDS deaths per 1,000 live births from the 1991 rate of 1.3 SIDS deaths per 1,000 live births. It is not currently known to what extent the decline in reported deaths is due to the adoption in 1991 of the new, more stringent, NICHD diagnostic criteria for SIDS. Nor is it known to what extent a decade of educating parents about

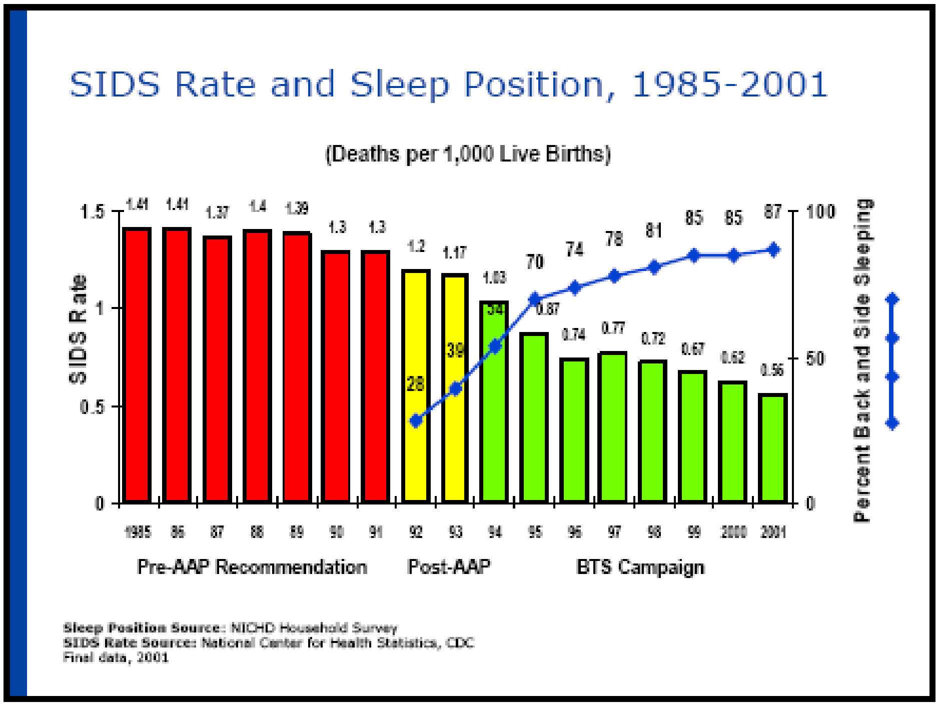

**FIGURE 1**. The reported rate for SIDS was already in decline prior to the issuance, in June 1992, of the AAP recommendation to place infants in the supine sleeping position and dropped 26% between 1989 and the start of the BTSC in 1994.

modifiable risk factors such as smoking, co-sleeping, and unsafe sleeping environments has contributed to the actual decline in SIDS deaths[12]. It cannot be known, therefore, to what extent altered sleep position is responsible for the reported decline in SIDS deaths.

In the U.S., evidence of an association between infant sleeping position and SIDS risk remains limited[13,14,15,16,17]. Only one study has been conducted completely after the BTSC was initiated[18]. However, this population-based, case-control study was limited by a low participation rate of 50% among eligible cases and 41% among eligible controls and a small sample size of 185 SIDS cases. Epidemiological analysis is also weakened by its reliance on retrospective questionnaires, which are subject to recall bias, and on interviews that often illicit responses intended only to please the interrogator. Furthermore, it is not possible to determine the relative importance or the combined effects of the many risk factors, in addition to sleeping position, that are thought to contribute to SIDS[19,20].

Clearly, the epidemiology of SIDS is confounded by cases where death may be due to other causes. Until the cause or causes of SIDS are found or a specific diagnostic test is developed, it cannot be known with certainty which infants succumb to “true” SIDS. Haas et al.[20] caution that the many overlapping epidemiological features of SIDS and non-SIDS infants illustrates the fallacy of assigning biological import only because of statistically significant differences. If a study sample is large enough, a small effect may be statistically significant even if the association is spurious because of a confounding variable[21]. Even risk factors with very large effects may not be important to individual cases if the disease is rare[21]. SIDS is a rare disease.

## The BTSC as Human Experimentation Rather than Confirmed Preventive Therapy

Neither the cause of SIDS nor a causal mechanism linking SIDS to the prone position have been documented despite more than a decade of intensive research. Multiple and unsubstantiated etiological theories abound. More than 49 causal mechanisms for SIDS have been recorded[22] and more than 38 epidemiological and biological risk factors proposed[19]. Various authors have disputed the major hypotheses (and their variants) that presume that a SIDS event results from abnormal cardiopulmonary regulation[23], arousal deficit[24,25], and asphyxia in general[26]. Until the etiology for SIDS has been validated and a causal relationship to the prone position has been established, scientific rigor requires that the BTSC be viewed as a large-scale human experiment rather than as a documented preventive therapy program.

It can be argued, of course, that a preventive program such as smoking cessation can be effective even though a causal mechanism has not yet been identified between smoking and lung cancer. However, the alternative to smoking, that is, not smoking, is not associated with harmful side effects. Such may not be the case with infant supine sleeping. Accordingly, it is appropriate to define and quantify clearly the relative risks and benefits of the BTSC and sleeping position intervention.

## Risks vs. Benefits of Infant Sleeping Position

In 2003, Hunt et al.[27] reported various health outcomes associated with infant sleep position and found that no identified symptoms such as cough, stuffy nose, spitting, vomiting, diarrhea, fever, or respiratory problems were significantly increased among nonprone sleepers. However, their data were retrieved from a 1995–1998 cohort study and were not examined for the risks of positional facial and head deformities (posterior *plagiocephaly*) and early developmental delay.

## Plagiocephaly

SIDS is a rare disease that claims the lives of approximately 0.5–3.0 infants per 1,000 births yearly in the developed countries of the world. By contrast, the incidence of positional facial and head deformities due to the supine position has increased dramatically since the BTSC and now affects 1 in every 60 live births[28]. Plagiocephaly sometimes requires physical therapy or head orthosis (helmet) and, if misdiagnosed as *craniosynostosis*, can result in unnecessary surgery. Plagiocephaly should not be dismissed lightly as merely “cosmetic”.

## Delayed Early Development

There are indications of a rapidly growing population of infants who show developmental abnormalities as a result of prolonged exposure to the supine position[29,30]. These infants do not have the opportunity to lift their heads when waking up or to explore the immediate environment around them. Poor development of antigravity extensor muscles leads to overall motor delays and difficulty acquiring key milestones such as rolling over, unsupported sitting, and crawling[29]. Although it is claimed that these developmental abnormalities are transient and tend to normalize in about 18 months[31], the evidence to support this viewpoint is scant and no long-term randomized clinical studies have been conducted.

## Sleeping Behavior

In the health outcomes study referred to above[27], it was also reported that sleep problems were not more frequent in infants sleeping supine compared to those sleeping prone. However, these observations conflict with an earlier report by this group that used the same 1995–1998 database[32]. In the earlier study, prone sleeping increased from 18% at 1 month to 29% at 3 months. The major reason given for changing the infants sleeping from supine at 1 month to prone at 3 months was that the infant "slept better or seemed to prefer that position". One possible explanation for the discrepancy is that the later study[27] excluded infants whose sleeping position may have changed after the first month due to sleeping problems or other adverse consequences of supine sleeping.

It is likely that prone is the normal sleeping position for infants[33] and offers the deepest, most restful sleep[34,35,36,37]. Because infants spend more time in sleep than children or adults, it is logical to assume that sleep is even more important for their rapidly developing nervous systems and for preserving the integrity of their sleep cycles[38].

## What is the Presumed Benefit of the Supine Sleeping Position?

Reference to Fig. 1 shows that the incidence of SIDS in the U.S. declined from 1.03 deaths per 1,000 live births in 1994, at the start of the BTSC, to 0.56 deaths per 1,000 live births in the year 2001. Proponents of the BTSC attribute these declining mortality statistics to altered sleeping position, and choose to characterize this effect as a dramatic 47% drop in the incidence of SIDS. However, these same data can also be presented as evidence that one infant death in 2,127 live births was prevented since the start of the BTSC if, in fact, placing infants in the supine rather than the prone position to sleep was the sole contributing factor.

These comments are not meant to diminish the importance or desirability of preventing even one SIDS event. Rather, they are intended to show the very low probability of a SIDS event occurring and the lower probability of it being prevented by placing an infant in the supine sleeping position.

However, the risks associated with the supine sleeping position, i.e., positional head deformities, delayed motor development, and disturbed sleep patterns, are substantive and relatively common. Finally, supine sleeping is not a guarantee against SIDS since some infants succumb in the supine position as well.

## CONCLUSION

In our enthusiasm to eradicate SIDS in the 0.2% of infants who are potential victims, we have tended to overlook other relative risks and benefits of the supine vs. the prone position in the 99.8% of infants who will not succumb to SIDS. An intuitive first reaction might be that the prevention of a SIDS event justifies virtually any intervention risk, since the unexpected death of an apparently healthy infant is one of the most devastating human tragedies in medicine. Indeed, if some 2,000 infant deaths are being prevented yearly by the BTSC, the campaign has made a substantial contribution to human welfare. However, this paper proposes that there are sound reasons for questioning these data and a sound basis for concern about the incompletely identified short- and long-term risks to child development of infant sleeping position intervention.

The hazards to public health that can result from failure to quantify a risk-benefit ratio accurately can be seen in the current controversy regarding hormonal replacement therapy in women[39]. The preponderance of epidemiological studies that strongly supported the protective effects of postmenopausal estrogen replacement against coronary heart disease[40], and the growing need for an effective means to treat and prevent heart disease in women, led to a nearly unshakable belief in the benefits of hormone therapy. As a consequence, many thousands of menopausal women may have received ineffectual treatment or possibly been exposed to an increased risk of heart disease.

Herrington[39] has proposed that while observational (epidemiological) studies may have important value for generating hypotheses, they should not be used to justify medical interventions for widespread use. Although the BTSC purports to recommend, rather than dictate, a preferred infant sleeping position, it has, in fact, set a standard of care that many pediatricians are reluctant to disregard[41]. Similarly, many parents are conflicted by their instinctive rejection of the supine infant sleeping position and their fear of charges of negligence in the event a SIDS death should occur.

The evidence in support of the premise of this paper, while compelling, is admittedly indirect and inconclusive. It is not the intention of the authors to discredit the BTSC. Rather, it is a plea to the pediatric research community to validate the nonprone infant sleeping position scientifically before it becomes inexorably imbedded in medical practice.

Until causal mechanisms are documented and risk/benefit ratios are identified, it may be more appropriate to regard the BTSC as investigational rather than as confirmed preventive therapy. In that context, the decision to place a child in the supine or prone position to sleep should be a personal and ethical choice that rests with the properly informed parent and not with the health care provider.

## ACKNOWLEDGMENTS

The authors are indebted to Drs. Coralee Thompson, Leland Green, Philip Bond and David Bergner for their critical reviews of the manuscript and insightful comments.

## BIOSKETCHES

**Ralph Pelligra, M.D**. is currently chief medical officer and Chair of the Institutional Review Board at NASA Ames Research Center. He has been instrumental in applying aerospace technology to a wide range of medical conditions, including clinical shock states and respiratory problems in brain injured children. He has published scientific and medical articles on diverse topics in The J. of Aerospace Medicine, JAMA, the Journal of Applied Physiology, Neuropediatrics, Emergency Medicine, and others. In 1996, Dr. Pelligra was inducted into the Space Technology Hall of Fame, sponsored by NASA and the U.S. Space Foundation. E-mail: ralph.pelligra-1@nasa.gov

**Glen Doman, DSc,** is the founder of The Institutes for the Achievement of Human Potential. He and The Institutes are famous for their pioneering work with brain-injured children and for their work in early development for well children. In addition to dealing intimately with more than 20,000 families over the last 50 years, he has strongly influenced millions of families through the book *What to Do About Your Brain-Injured Child* and the creation of the groundbreaking Gentle Revolution Series of books and materials that teach parents how to teach their babies at home. Glenn Doman has lived with, studied, or worked with children in more than 100 nations, ranging from the most civilized to the most primitive. E-mail: vicedirector@iahp.org

**Gerry Leisman, MD, PhD,** is the Dr. Ted Carrick Professor of Human Factors at the Institute for Clinical Ergonomics, Rehabilitation, and Applied Neuroscience in the School of Engineering Technologies at the State University of New York, College at Farmingdale and Affiliate Professor at the University of Haifa in Israel. He was elected Fellow of the American Psychological Society in 1990 and Life Fellow of the American College of Forensic Examination-International in 1994. He is the author of numerous studies, textbooks, and patents in rehabilitation sciences, mathematical modeling of the nervous system, human memory, development of motor and cognitive function, and in cognitive neuroscience. E-mail: leismag@farmingdale.edu
